# Supplementary material for: Single-cell transcriptome atlas and chromatin accessibility landscape reveal differentiation trajectories in the rice root
Source: Nat Commun. 2021 Apr 6;12:2053. doi: 10.1038/s41467-021-22352-4 (PMC8024345; doi:10.1038/s41467-021-22352-4)
Supplement: Supplementary file 1 — Supplementary information [file 41467_2021_22352_MOESM1_ESM.pdf]

Supplementary Note 1

Rice also develops crown roots. On the basis of on their origins, crown roots can be divided into two categories: embryonic crown roots emerging from the coleoptile node and postembryonic crown roots differentiating from the nodes of the main stem and tillers. The radicle and crown roots can branch to generate two types of secondary roots: large lateral roots that are geotropic and indeterminate, and small lateral roots that are ageotropic and determinate.

Previous studies revealed common features in the molecular regulation of root formation in cereals and Arabidopsis[1-3]. For example, auxin plays a conserved and critical role in the maintenance of the QC. A stabilizing mutation in domain II of rice AUX/IAA protein, OsIAA23, leads to a loss of QC identity during postembryonic development[4]. *WUSCHEL-RELATED HOMEOBOX5* (*WOX5*) is exclusively expressed in the Arabidopsis QC[5]. Similarly, a rice WOX5-type homeobox gene, named quiescent-center-specific homeobox (*QHB*), is specifically expressed in the central cells of QC[6]. Moreover, the CLAVATA3 (CLV3)/ENDOSPERM SURROUNDING REGION (ESR)-related CLE peptides have been found to inhibit the expression of *WOX5* or *QHB* in both Arabidopsis and rice[7-10]. The lysigenous aerenchyma is an internal gas space generated by programmed cell death and lysis of cortical cells[11,12]. In rice, aerenchyma can be constitutively formed or induced under oxygen-deficient conditions[13]. Thus, the unique radial anatomy of the rice roots, i.e. the formation of exodermis, sclerenchyma and aerenchyma, reflects an adaptation to semiaquatic life conditions.

A number of mutants involved in rice root development have been identified using a forward genetics approach[14,15]. However, only a limited number of these genes have been functionally characterized. The discoveries of new root developmental genes and construction of a comprehensive networks for root cell-specific functions and interactions require sophisticated mutant screens, reverse genetics, and the development of a root tissue or cell-specific transcriptome atlas. For instance, a population of rice UAS-GAL4-GFP lines was used for the isolation of more than 100 lines displaying a cell-specific pattern of expression in roots[16]. In addition, a rice seedling transcriptome atlas which includes 40 cell types has been produced by laser capture microdissection[17]. However, this dataset is limited in scope to a few root cell types or encompass zones or tissues combining multiple cell types.

Supplementary Note 2

Here we described how we annotated cell clusters of rice radicles.

Epidermis

Clusters 1, 4 and 9 belonged to the epidermis/root hair population (Fig. 1a). These clusters were separated from other cell clusters on the UMAP plot (Fig. 1a), suggesting a unique transcriptome signature. *ROOT HAIRLESS1* (*OsRHL1*), a key regulator of rice root hair development, was highly expressed in cluster 4 (Fig. 1d; Supplementary Fig. 9)[18,19]. RNA *in situ* hybridization assays further reveled that the transcripts of the cluster 1 and 9-specific genes *Os03g0155900* and *Os10g0454200*, accumulated to high levels in the epidermis (Supplementary Fig. 10a, b). In support of these findings, rice *cellulose synthase-like D1* (*OsCSLD1*) and *Sec14-nodulin domain protein* (*OsSNDP1*) genes, which play important roles in root hair morphogenesis[20,21], were overrepresented in cluster 4 (Fig. 1d; Supplementary Fig. 9). Likewise, transcripts of the phosphate (Pi) transporter gene *OsPht1;2* (*OsPT2*), which is predominantly expressed in epidermal cells of primary roots[22], were readily detected in clusters 1 and 4 (Fig. 1d; Supplementary Fig. 9). Moreover, the epidermis/exodermis-specific gene *purple acid phosphatases10c* (*OsPAP10c*) was also predominantly expressed in clusters 1 and 9 (Fig. 1d; Supplementary Fig. 9)[23].

Intriguingly, cell cluster specific gene analysis revealed a strong enrichment of transporter genes in the epidermal clusters (Supplementary Fig. 11; Supplementary Data 2). We found, for example, that two potassium transporter genes (*OsHAK11* and *OsHKT1;1*) were highly expressed in clusters 1 and 4 (Supplementary Fig. 11; Supplementary Data 2)[24,25]. In addition, all three clusters had abundant transcripts for the nitrate transporter (*OsNAR2.1*, *NRT2.1*, and *OsNRT2.2*) and ammonium transporter (*OsAMT1;2* and *OsAMT3;2*) genes (Supplementary Fig. 11; Supplementary Data 2)[26,27]. Moreover, we observed high levels of transcripts for the genes involved in calcium, iron, sulfur, and water transport (Supplementary Fig. 11). Thus, these results imply that the epidermis, including root hairs, plays an important role in nutrient uptake and ion homeostasis in rice.

Ground tissue

The rice ground tissue is made up of four cell layers, namely the exodermis, sclerenchyma, cortex and endodermis, from outside-in (Fig. 1). Clusters 3 and 8 were assigned as exodermis cells because the

cluster-specific gene *Os04g0125700* was exclusively expressed in the exodermis (Supplementary Fig. 10c). Clusters 6 and 0 were assigned as the sclerenchyma cell layer and cortex, respectively, as determined by the specific accumulation transcripts of the cluster-specific genes, *Os08g0115800*, *Os01g914100* and *Os03g135700*, in the corresponding cell layers (Supplementary Fig. 10d-f).

As mentioned earlier, plants have developed several strategies to control nutrient uptake for optimal growth. One such strategy is the formation of a lignin-based diffusion barrier called the Casparian strip (CS) at the exodermis and endodermis of rice roots. In Arabidopsis, the CS is formed by the concerted action of localized NADPH oxidase and peroxidases through the action of Casparian strip domain proteins (CASPs)[28]. Interestingly, we found that the genes encoding secreted (type III) peroxidases (*prx4*, *prx27*, *prx34* and *prx74*) were overrepresented not only in the sclerenchyma cell layer (clusters 6) but also in exodermal cells (clusters 3 and 8) (Supplementary Fig. 12; Supplementary Data 2). Therefore, these results imply that, in contrast to the endodermis of Arabidopsis, the formation of CS in rice sclerenchyma cell layer requires coordination of two adjacent cell layers: the secreted peroxidases from exodermis may also contribute to H_2O_2-dependent lignin polymerization in the sclerenchyma cell layer.

Two cell clusters (2 and 13) were linked to endodermal cells. *Casparian strip domain protein1* (*OsCASP1*), which is required for endodermal CS formation in Arabidopsis[29], was highly enriched in cluster 13 (Fig. 1d; Supplementary Fig. 9). In contrast, *Low silicon rice1* (*Lsi1*), which encodes a silicon transporter localized at the distal side of endodermis[30], was predominantly expressed in cluster 2 (Supplementary Fig. 9). The expression of *Os10g155100*, the marker gene for cluster 13, was restricted to endodermal cells of the maturation zone (Supplementary Fig. 10g). Thus, these results suggest that rice root endodermal cells are not homogenous; endodermal cells involved in CS formation (cluster 13) feature a distinct transcriptome. Consistently, clusters 2 and 13 were topologically separated on the UMAP. The association of cluster 13 with cluster 5 suggests that this cluster may retain some meristematic activity. Similar to the enrichment of secreted peroxidases in the sclerenchyma cell layer, the elevated expression of peroxidase genes, including as *prx5*, *prx54*, *prx86*, *prx111* and *prx112*, was observed in clusters 2 and 13 (Supplementary Fig. 12; Supplementary Data 2).

Pericycle and vascular tissues

We annotated cluster 15 as root pericycle based on the reported expression patterns of the citrate transporter genes *OsFRDL1* and *Os07g0634400* (Fig. 1d; Supplementary Fig. 9; Supplementary Fig. 10h)[31]. Interestingly, the expression of an outward-rectifying Shaker-like potassium channel gene *OsSKOR* and a putative auxin transport gene *OsCOLE1-INTERACTING PROTEIN* (*OsCLIP*) was also detected in this cluster (Fig. 1D; Supplementary Fig. 9)[32,33].

Cluster 17 was designated as phloem based on the strong expression of the phloem-specific gene *FT-INTERACTING PROTEIN1* (*OsFTIP*) (Fig. 1d; Supplementary Fig. 9)[34]. Clusters 12 and 16 were assigned as xylem because three marker genes, *Os01g0750300*, *Os08g0489300* and *Os07g0638500*, were strongly expressed in this tissue as revealed by *in situ* hybridization assays (Supplementary Fig. 10i-k). In line with heavy deposition of lignin in the tracheary elements of xylem, genes related to the lignin monomer biosynthetic genes such as *phenylalanine ammonia-lyase* (*PAL*), *4-coumarate:CoA ligase* (*4CL*), *cinnamyl alcohol dehydrogenase* (*CAD*) and *caffeic acid O-methyl transferase* (*COMT*) and *cinnamoyl-CoA reductase* (*CCR*) were highly enriched in cluster 12 (Supplementary Data 2)[35]. The assignment of cluster 16 as xylem was further confirmed by RNA *in situ* hybridization assays of two marker genes, a Class III HD-Zip gene *OsHB4* which is expressed in the xylem tissue of vascular bundles, and *SECONDARY WALL NAC DOMAIN PROTEIN6* (*OsSWN6*) which encodes one of the NAC transcription factors involved in secondary wall formation (Supplementary Fig. 9)[36-38].

We related clusters 7 and 10 to other cell types in the vascular tissues. Three previously identified vascular genes including *SALT TOLERANCE RECEPTOR-LIKE CYTOPLASMIC KINASE1* (*STRK1*), *SALT-RESPONSIVE ERF1* (*SERF1*) and *ACTIN DEPOLYMERIZING FACTOR3* (*OsADF3*) were preferentially expressed in these two clusters (Supplementary Fig. 9)[39-41].

Meristematic cells

Each cell type in the root tip is composed of dividing cells (i.e. transit-amplifying cells) and differentiated cells. Since the unsupervised cell clustering method sorts all the single cells into distinct cell clusters by the similarities in transcriptome, all the dividing cells in the root tip tend to group together, regardless which cell types they belong to. Clusters 5, 11, 18 and 19 consisted of root meristem cell populations. The genes involved in DNA synthesis such as *histone H2A* (*H2A*), *H2B.2*, *H3*, *H3R-11* were highly expressed in cluster 18, whereas transcripts of *CyclinB1;1* (*cycB1;1*), *cycB2;2* and

cyclin-dependent kinase B2;1 (*OsCDKB2;1*) accumulated in cluster 19 (Fig. 1d; Supplementary Fig. 9). Therefore, we designated clusters 18 and 19 as representing cells in the S and G2 phases, respectively. The cells in clusters 5 and 11 showed an enrichment of the genes encoding ribosomal proteins and biological processes related to translation (Supplementary Data 2). In yeast and mammals, translation rates are robust in the G1 phase of the cell cycle but are low during mitosis[42]. Thus, we concluded that the cells in clusters 5 and 11 represented the cells in the G0/G1 phase as well as those undergoing differentiation, also known as transit-amplifying cells[43]. Consistent with the above annotations, the transcripts of *QUIESCENT-CENTER-SPECIFIC HOMEOBOX* (*QHB*) gene, a putative orthologue of Arabidopsis root stem-cell niche organizer *WUSCHEL (WUS)-RELATED HOMEOBOX5* (*WOX5*)[6,7], was detected in cluster 5 (Supplementary Fig. 9). Moreover, *in situ* hybridization assays revealed most of the marker genes (*Os01g0273100*, *Os02g0805200*, *Os03g0279200*, *Os04g0496300*, *Os05g438700* and *Os08g0490900*) of clusters 5, 18 and 19 exhibited sporadic expression patterns in the RAM (Supplementary Fig. 10m-r).

Os03g0247200, the marker gene for cluster 14, was specifically expressed in the root cap junction, a cell layer with thick cell wall between root cap and the proximal root meristem (Supplementary Fig. 10l)[44]. As such, we assigned cluster 14 as root cap junction. Cluster 20 could not be annotated because of the lack of known marker genes. We did not annotate any cell clusters corresponding to the root cap, probably due to the fact that root cap cells are largely resistant to cell wall digestion during protoplast preparations.

Supplementary Note 3

We examined the expression pattern of known transcription factors genes involved in the root development on the UMAP. *WUSCHEL-RELATED HOMEOBOX5* (*WOX5*) is exclusively expressed in the Arabidopsis QC[5]. *OsWOX5* was expressed in meristematic cell clusters (Supplementary Fig. 13a). However, due to low number of WOX5 positive cells, we could not faithfully annotate a cell cluster corresponding to rice QC in our atlas. It has been shown that two transcription factors SCARECROW (SCR) and SHORT-ROOT (SHR) play important roles in ground tissue differentiation in Arabidopsis[45]. Consistently, both *OsSCR1* and *OsSCR2* were expressed in endodermal cluster (cluster 13), whereas *OsSCR2* transcripts could be also detected in the cortex cell cluster (cluster 0). Similarly, *OsSHR1* and *OsSHR2* were predominantly expressed in meristematic and endodermal clusters (Supplementary Fig. 13a). The transition from cell division to cell expansion and differentiation in distinct root developmental zones is guided by a gradient distribution of a group of transcription factors named PLETHORA (PLT)[46]. Four rice *PLT* genes (*OsPLT2*, *OsPLT3*, *OsPLT5*, and *OsPLT9*) were expressed in the root meristematic cell clusters (Supplementary Fig. 13a).

Because auxin has been implicated in root development in Arabidopsis, we then probed the expression of the genes involved in auxin transport and signaling transduction including *PIN-FORMED* (*PIN*), *AUXIN RESPONSE FACTOR* (*ARF*) and AUX/IAA[47-50]. As shown in Supplementary Fig. 13b, while *OsPIN1C* (*Os11g0137000*) and *OsPIN1D* (*Os12g0133800*) were predominantly expressed in the meristematic cell clusters, *OsPIN10B* (*Os05g0576900*) was broadly expressed in the root tip. Among all the rice *ARFs*, the transcripts of *ARF2*, *ARF7*, *ARF9* and *ARF22* could be detected in the epidermal cells (Supplementary Fig. 13b). On the contrary, *ARF16* was highly expressed in the RAM, endodermis and vascular tissues. *IAA17* and *IAA12* conferred highest and broadest expression within all the AUX/IAA genes. *IAA2* was specifically expressed in one of the endodermal cell cluster (cluster 13).

Supplementary Figures

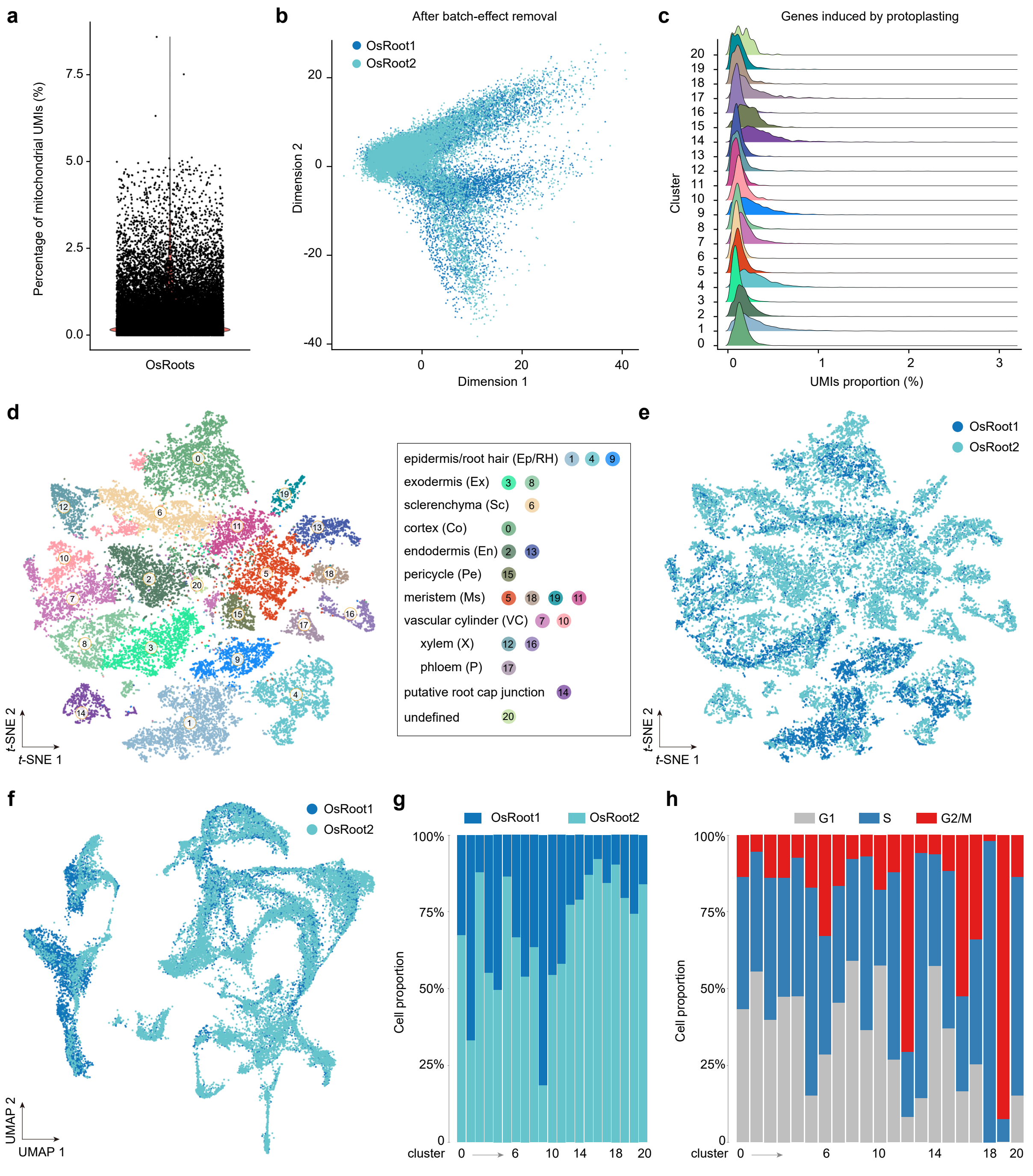

Supplementary Fig. 1 | Summary the rice root scRNA-seq.

a, The percentage of mitochondrial UMIs in each cell. **b,** The scatterplot showing top two dimensions of harmony. The batch-effect from two biological replicates was removed by the Harmony algorithm[51]. **c,** A ridge plot showing the UMIs proportion of protoplasting genes [52]. Please note that protoplasting genes were not enriched in cell clusters. **d,** *t*-SNE visualization of 21 cell clusters of rice radicles. Each dot denotes a single cell. Colors denote corresponding cell clusters as in **Fig. 1a**. **e,f,** *t*-SNE (**e**) and

UMAP (**f**) visualization of two biological replicates. **g,** Proportion of the cells from two biological replicates in each cell cluster. h, Proportion of inferred cell cycle phases (G1, S and G2/M) in each cell cluster. The cell cycle phase was inferred from the transcriptome using the "CellCycleScoring" function in Seurat. Please note that meristematic cell populations (C5, C11, C18 and C19) were predominant in the G2/M and S phases, whereas differentiated cells (C1, C8, C10 and C14) were mainly in the G1 phase. **f,** The perspective view of the 3D UMAP at different angles. Cluster names and colors are the same as in **Fig. 1a**.

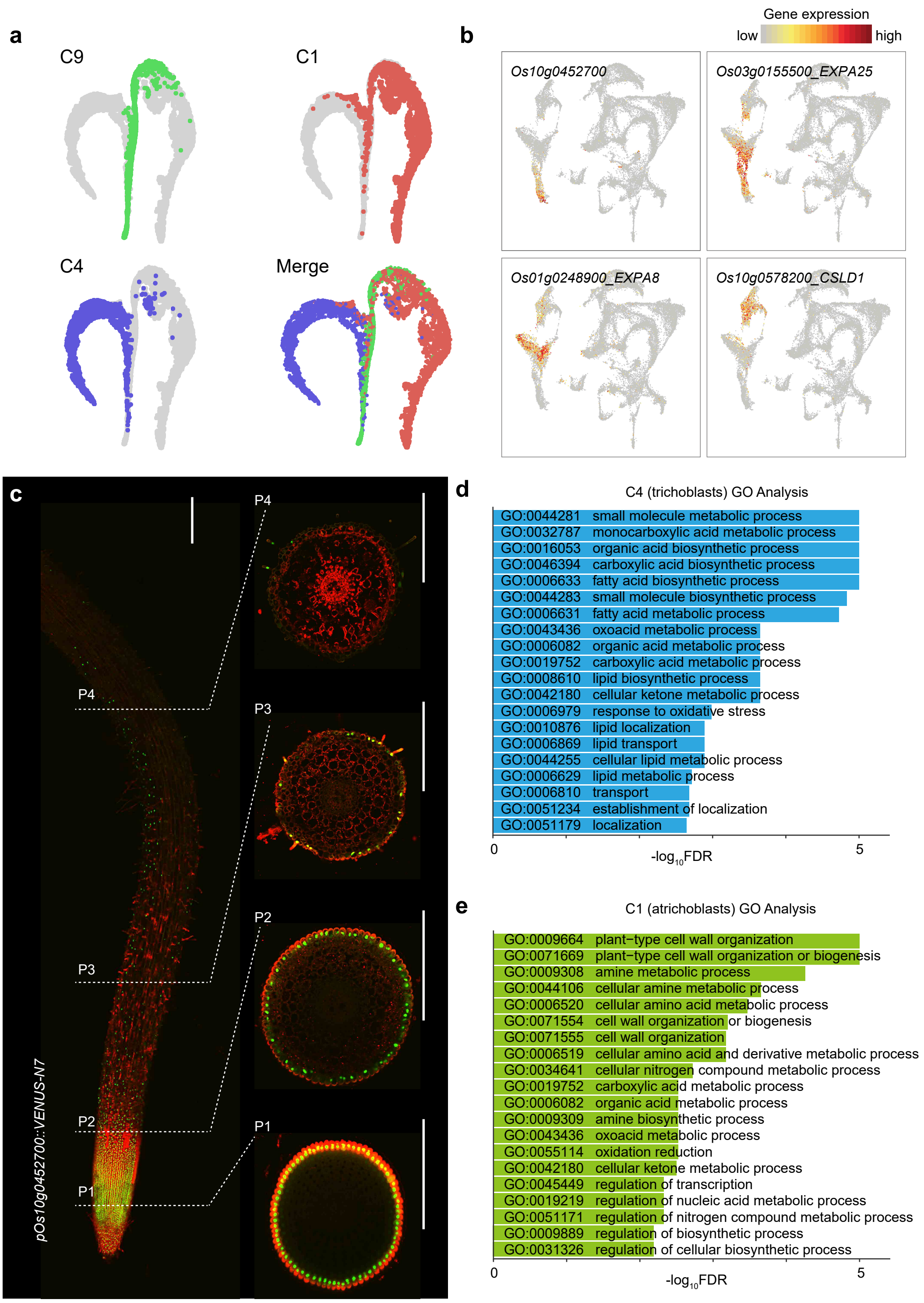

Supplementary Fig. 2 | Differentiation trajectories of trichoblasts and atrichoblasts.

a, *t*-SNE map showing the epidermal cell populations. Colored by clusters: C1, red; C4, purple; C9, green. **b**, UMAP plot showing the expression pattern of selected marker genes for clusters 1, 4 and 9. **c,** Expression of *pOs10g10g0452700::VENUS-N7* reporter (green) in rice radicles. the positions (P1 to P4) for transverse sections are shown. Six individual plants were observed. Scale bar, 200 µm. **d**,**e**, Top

20 GO terms of trichoblasts (C4) and atrichoblasts (C1). The distinct GO enrichments of trichoblasts and atrichoblasts suggest that they are functionally specialized. $-\text{Log}_{10}$FDR for each item is given.

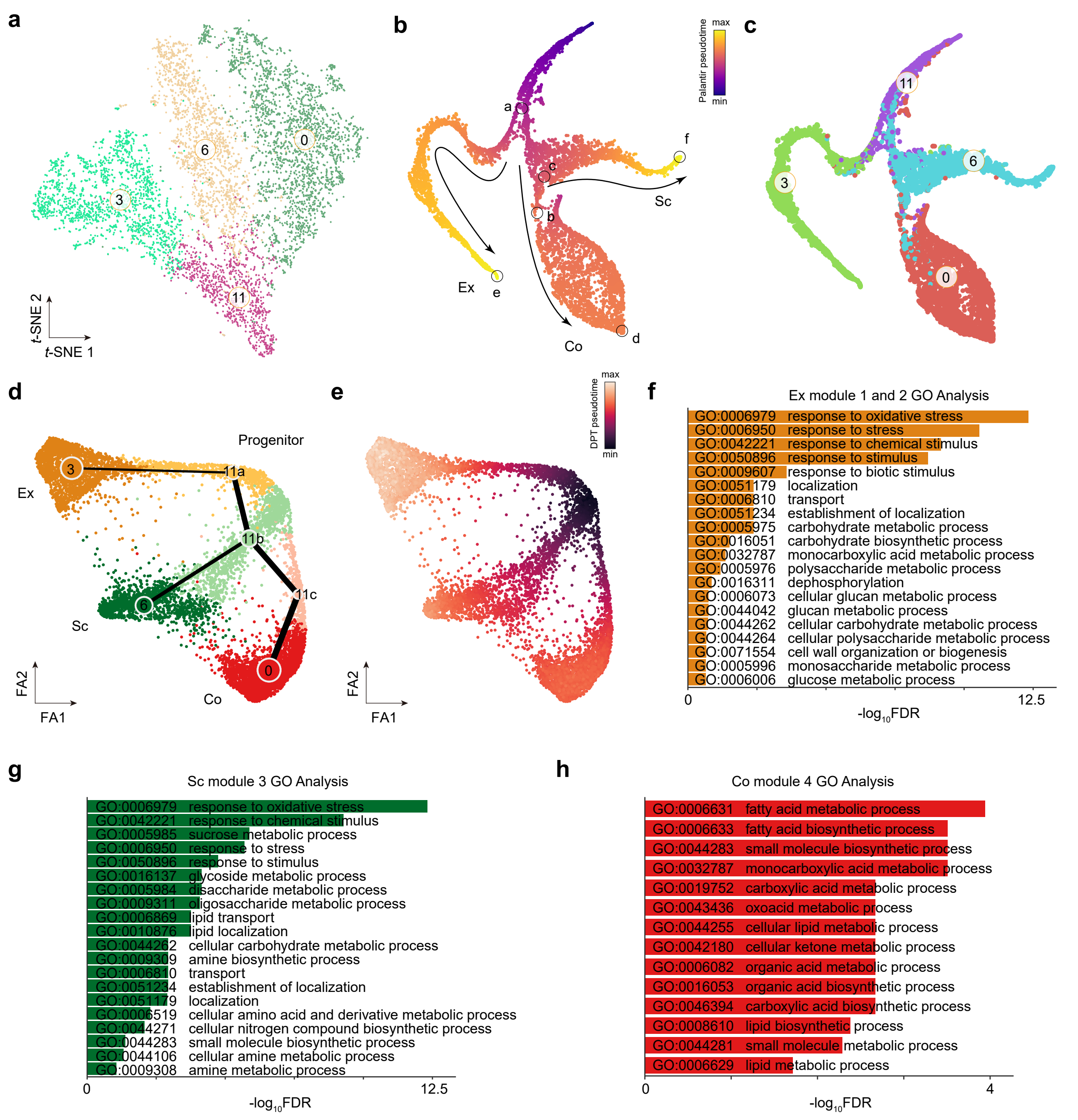

Supplementary Fig. 3 | Differentiation trajectories of ground tissues.

a, *t*-SNE plot showing the topology of clusters 0, 3, 6 and 11. Please note that C3, C6 and C0 are all connected to C11. **b**,**c**, *t*-SNE plot showing clusters 0, 3, 6 and 11. Colored by Palantir pseudotime (**b**) or cell cluster (**c**). Three differentiation trajectories toward exodermis (Ex, C3), cortex (Co, C0) and sclerenchyma layer (Sc, C6) are shown. a to f (black circles), six nodes used for quantification of branch probabilities as in **Fig. 3d**. **d**,**e**, ForceAtlas2 layout showing differentiation path of ground tissues. Colored by cell type (**d**) or DPT pseudotime (**e**). Ex, orange; Co, red; Sc, green. The cells in cluster 11 can be separated into three progenitor cell clusters, named to 11a, 11b and 11c. **f-h**, Top 20 GO terms of representative gene modules 1 and 2 for Ex, module 3 for Sc and module 4 for Co in **Fig. 3h-j**. -

$Log_{10}FDR$ for each item is given.

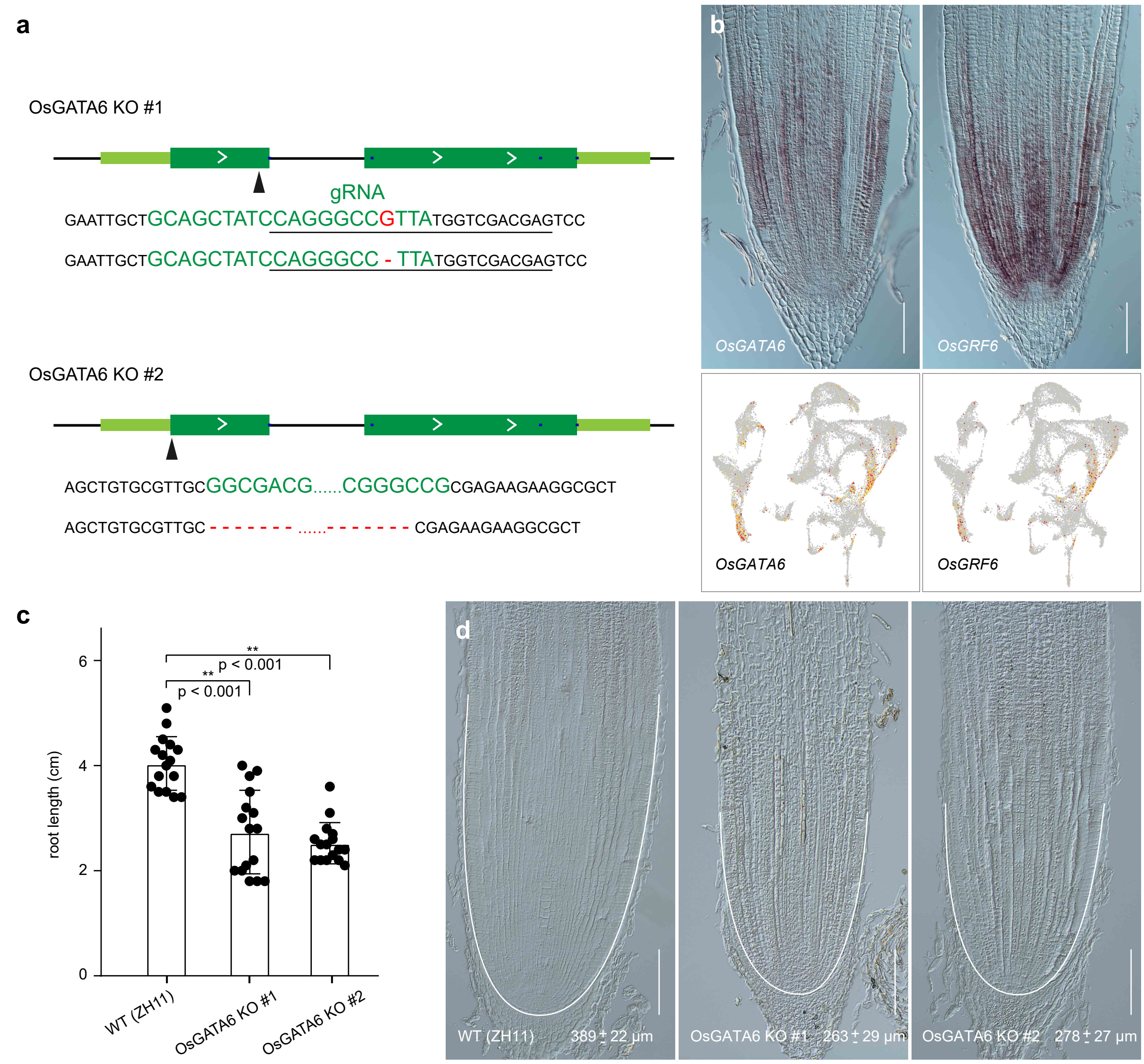

Supplementary Fig. 4 | Characterization of rice *OsGATA6* mutants.

a, Schematic presentation of generation of the *OsGATA6* mutants (KO #1 and #2). The target site (arrowhead), sgRNA sequence (green), the mutated nucleotide (red), and Sanger sequencing results (below) are shown. **b**, Expression pattern of *OsGATA6* and *OsGRF6* in the rice root tip revealed by RNA *in situ* hybridization assays (top panels) and UMAP plot (bottom panels). Eight individual plants for each gene were observed. Scale bar, 100 μm. **c**, The root length of WT (ZH11) and *OsGATA6* mutants (KO #1 and #2). Plants were grown on 1/2 MS mediums in long days at 29°C for 4 days. One-way ANOVA was performed by the Turkey's multiple comparisons test, $^{**}p < 0.01$. $n = 16$ individual plants. **d**, RAM size of wild type (left) and *OsGATA6* (right) mutants. Six individual plants were observed. One representative root for each genotype is shown. Line marks the RAM. The size of RAM is given. Scale bar, 100 μm.

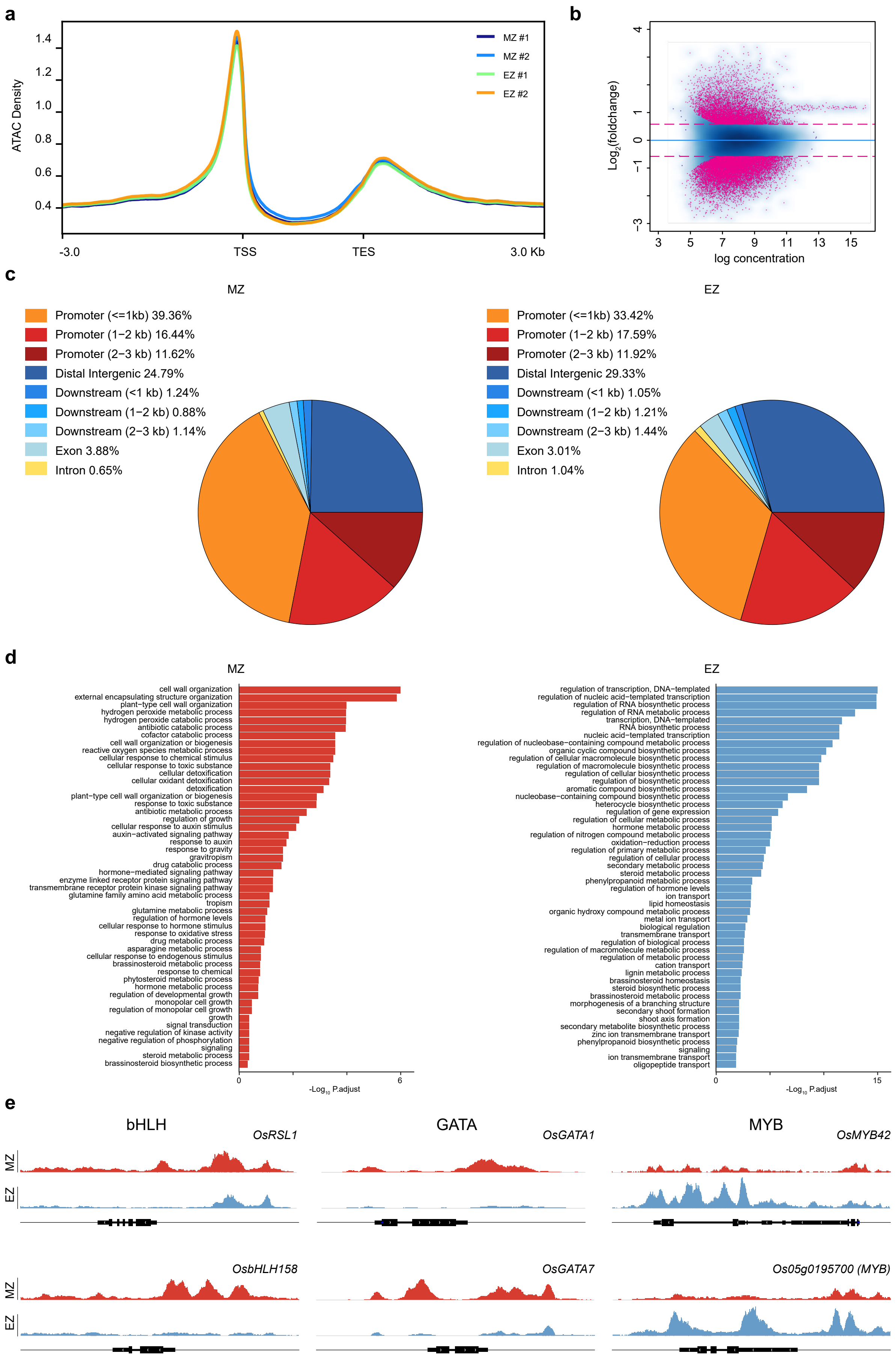
a
ATAC Density
1.4
1.2
1.0
0.8
0.6
0.4
MZ #1
MZ #2
EZ #1
EZ #2
-3.0
TSS
TES
3.0 Kb
b
Log_2(foldchange)
4
1
0
−1
−3
3
5
7
9
11
13
15
log concentration
c
MZ
Promoter (<=1kb) 39.36%
Promoter (1−2 kb) 16.44%
Promoter (2−3 kb) 11.62%
Distal Intergenic 24.79%
Downstream (<1 kb) 1.24%
Downstream (1−2 kb) 0.88%
Downstream (2−3 kb) 1.14%
Exon 3.88%
Intron 0.65%
EZ
Promoter (<=1kb) 33.42%
Promoter (1−2 kb) 17.59%
Promoter (2−3 kb) 11.92%
Distal Intergenic 29.33%
Downstream (<1 kb) 1.05%
Downstream (1−2 kb) 1.21%
Downstream (2−3 kb) 1.44%
Exon 3.01%
Intron 1.04%
d
MZ
cell wall organization
external encapsulating structure organization
plant−type cell wall organization
hydrogen peroxide metabolic process
hydrogen peroxide catabolic process
antibiotic catabolic process
cofactor catabolic process
cell wall organization or biogenesis
reactive oxygen species metabolic process
cellular response to chemical stimulus
cellular response to toxic substance
cellular detoxification
cellular oxidant detoxification
detoxification
plant−type cell wall organization or biogenesis
response to toxic substance
antibiotic metabolic process
regulation of growth
cellular response to auxin stimulus
auxin−activated signaling pathway
response to auxin
response to gravity
gravitropism
drug catabolic process
hormone−mediated signaling pathway
enzyme linked receptor protein signaling pathway
transmembrane receptor protein kinase signaling pathway
glutamine family amino acid metabolic process
tropism
glutamine metabolic process
regulation of hormone levels
cellular response to hormone stimulus
response to oxidative stress
drug metabolic process
asparagine metabolic process
cellular response to endogenous stimulus
brassinosteroid metabolic process
response to chemical
phytosteroid metabolic process
hormone metabolic process
regulation of developmental growth
monopolar cell growth
regulation of monopolar cell growth
growth
signal transduction
negative regulation of kinase activity
negative regulation of phosphorylation
signaling
steroid metabolic process
brassinosteroid biosynthetic process
0
6
$-Log_{10}$ P.adjust
EZ
regulation of transcription, DNA−templated
regulation of nucleic acid−templated transcription
regulation of RNA biosynthetic process
regulation of RNA metabolic process
transcription, DNA−templated
RNA biosynthetic process
nucleic acid−templated transcription
regulation of nucleobase−containing compound metabolic process
organic cyclic compound biosynthetic process
regulation of cellular macromolecule biosynthetic process
regulation of macromolecule biosynthetic process
regulation of cellular biosynthetic process
regulation of biosynthetic process
aromatic compound biosynthetic process
nucleobase−containing compound biosynthetic process
heterocycle biosynthetic process
regulation of gene expression
regulation of cellular metabolic process
hormone metabolic process
regulation of nitrogen compound metabolic process
oxidation−reduction process
regulation of primary metabolic process
regulation of cellular process
secondary metabolic process
steroid metabolic process
phenylpropanoid metabolic process
regulation of hormone levels
ion transport
lipid homeostasis
organic hydroxy compound metabolic process
metal ion transport
biological regulation
transmembrane transport
regulation of biological process
regulation of macromolecule metabolic process
regulation of metabolic process
cation transport
lignin metabolic process
brassinosteroid homeostasis
steroid biosynthetic process
brassinosteroid metabolic process
morphogenesis of a branching structure
secondary shoot formation
shoot axis formation
secondary metabolite biosynthetic process
zinc ion transmembrane transport
phenylpropanoid biosynthetic process
signaling
ion transmembrane transport
oligopeptide transport
0
15
$-Log_{10}$ P.adjust
e
bHLH
OsRSL1
GATA
OsGATA1
MYB
OsMYB42
MZ
EZ
OsbHLH158
OsGATA7
Os05g0195700 (MYB)
MZ
EZ

Supplementary Fig. 5 | Analysis of ATAC-seq data.

a, The genome-wide distribution of all ATAC-seq peaks among each sample. Window size: gene body ± 3.0 kb. TSS, transcription start sites; TES, transcription end site. **b**, MA plots showing fold-change of differentially accessible peaks among the MZ and EZ. Blue, distribution of constitutive peaks; pink dots, individual differential peaks. **c**, Location of ATAC-seq peaks in the MZ (left) and EZ (right) samples. All the differential peaks can be sorted into nine categories based on their relative locations in the Nipponbare genome (shown in different colors). The percentage of each category is given. Please note that 39.36% peaks in the MZ resided in the promoter (<= 1 kb) region, in comparison to 33.42% in the EZ; In contrast, the EZ sample had a higher accessibility in the distal intergenic regions (24.79% in the MZ vs 29.33% in the EZ). This difference suggests a dynamics in chromatin accessibilities along cell differentiation from the MZ to EZ. **d**, GO analyses of differentially accessible genes. The selected 50 enriched GO biological processes are indicated. $-\mathrm{Log}_{10}$(p.adj) is given. **e**, Representative ATAC-seq tracks for bHLH, GATA and MYB family transcription factor genes. The genomic loci are shown, and the representative genes are highlighted in black. Two representative genes for each transcription factor family are shown.

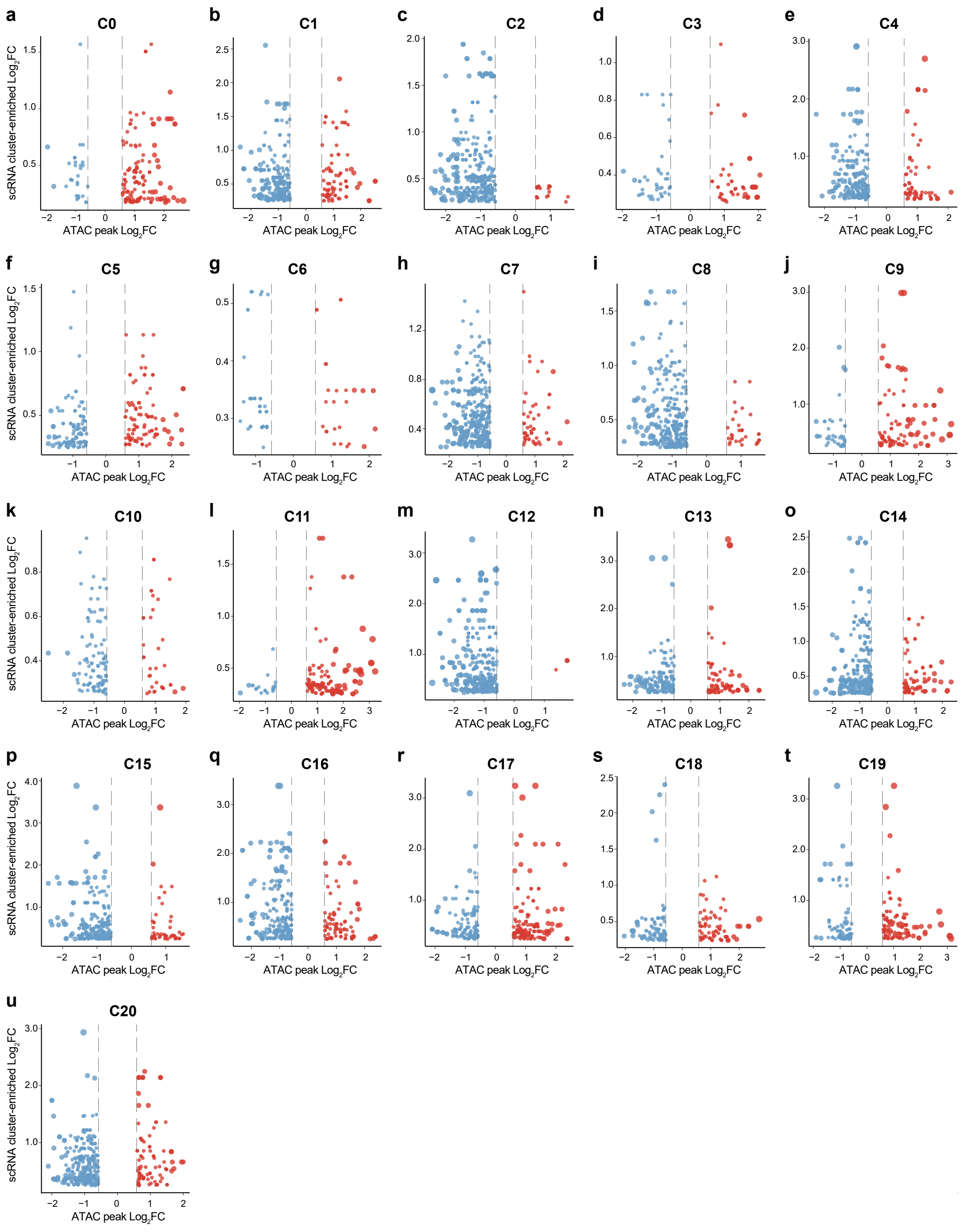

Supplementary Fig. 6 | Integrative analysis of ATAC-seq and scRNA-seq data.

The accessibilities of cluster-specific genes are shown. Each dot represents one cluster- specific gene. Red, highly accessible in the MZ; Blue, highly accessible in the EZ. The genes associated with differential peaks (Log_2(foldchange) >= 0.58 or <= -0.58) are shown. Twenty-one cell clusters (C0 to

C21, **a-u**) identified by scRNA-seq (**Fig. 1a**) are shown. Please note that the accessibilities of cluster-specific gene are largely correlated with the nature of cell type. For example, the genes in cluster 9 (meristematic cells for epidermal cells) exhibited higher accessibility in the MZ than in the EZ. In contrast, the genes enriched in clusters 1 and 4 (root hair and non-root hair cell clusters) are highly accessible in the EZ, rather than in the MZ.

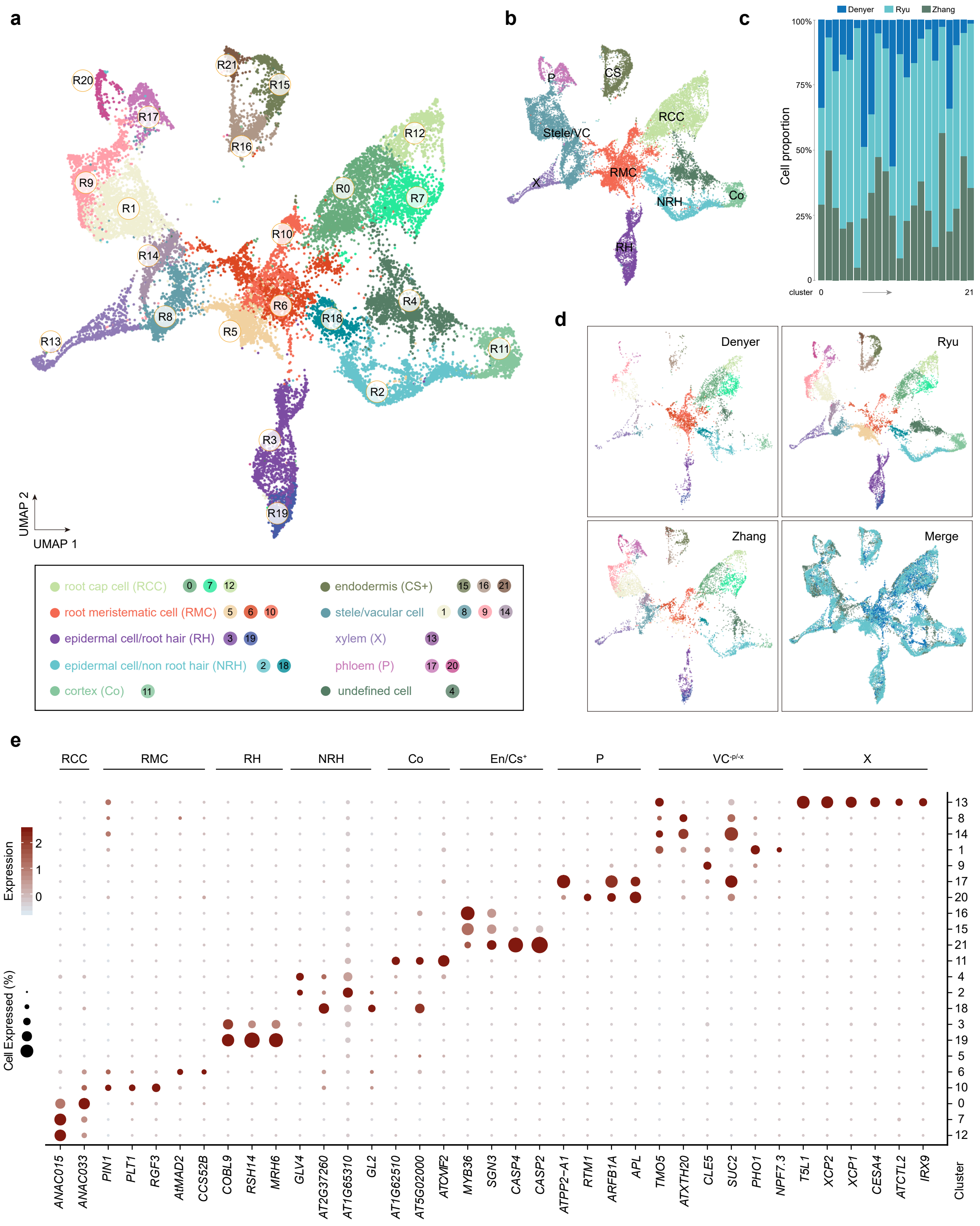

Supplementary Fig. 7 | Integrative analysis of Arabidopsis root scRNA-seq datasets.

a, UMAP plot showing Arabidopsis root cell clusters. Three published datasets were merged and re-clustered (Methods). In total, 22 cell clusters (R0 to R21) were identified. The assignment of each cluster is given. Each dot denotes a single cell. Colors denote corresponding cell clusters. **b**, Broad Arabidopsis root cell populations. Co, cortex; NRH, non-root hair cells; P, phloem; RCC, root cap cells;

RH, root hairs; RMC, root meristematic cells; Stele/VC, stele/vascular tissues; X, xylem. **c**, Proportion of the cells from three published Arabidopsis root scRNA-seq datasets (Denyer, Ryu and Zhang) in each cluster. **d**, UMAP plot showing the distribution of the cells from three published Arabidopsis root scRNA-seq datasets (Denyer, Ryu and Zhang). **e**, Expression pattern of representative cluster-specific marker genes. Cluster number (R0 to R21) is given on the right. En/Cs^{+}, endodermis/endodermis with Casparian strip; VC$^{-P/-X}$, vascular tissue without phloem and xylem. Dot diameter, proportion of cluster cells expressing a given gene. The full names and references for these selected genes are given in Supplementary Data 3.

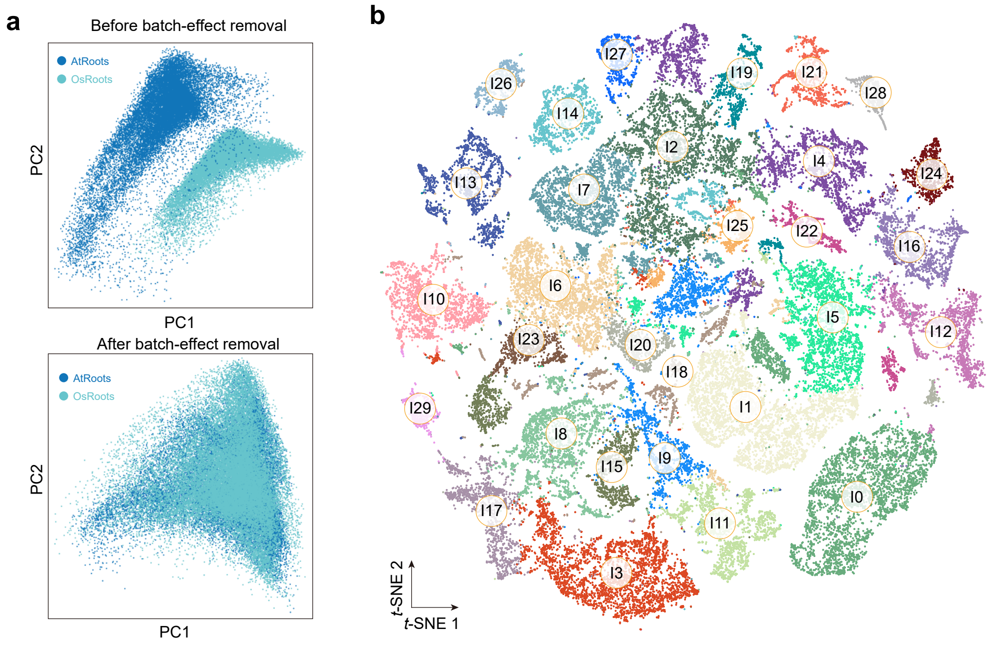

Supplementary Fig. 8 | Comparative analysis of rice and Arabidopsis root scRNA-seq datasets. **a**, Evaluation of batch effects across species. The top two PCs showing the batch-effects between rice and Arabidopsis scRNA-seq datasets (top). After dataset integration using Canonical Correlation Analysis (CCA) in Seurat, this effect was eliminated (bottom). **b**, *t*-SNE plot showing 30 super root cell clusters (I0 to I29) revealed by the integration of rice and Arabidopsis scRNA-seq datasets. Each dot denotes a single cell. Colors denote corresponding cell clusters.

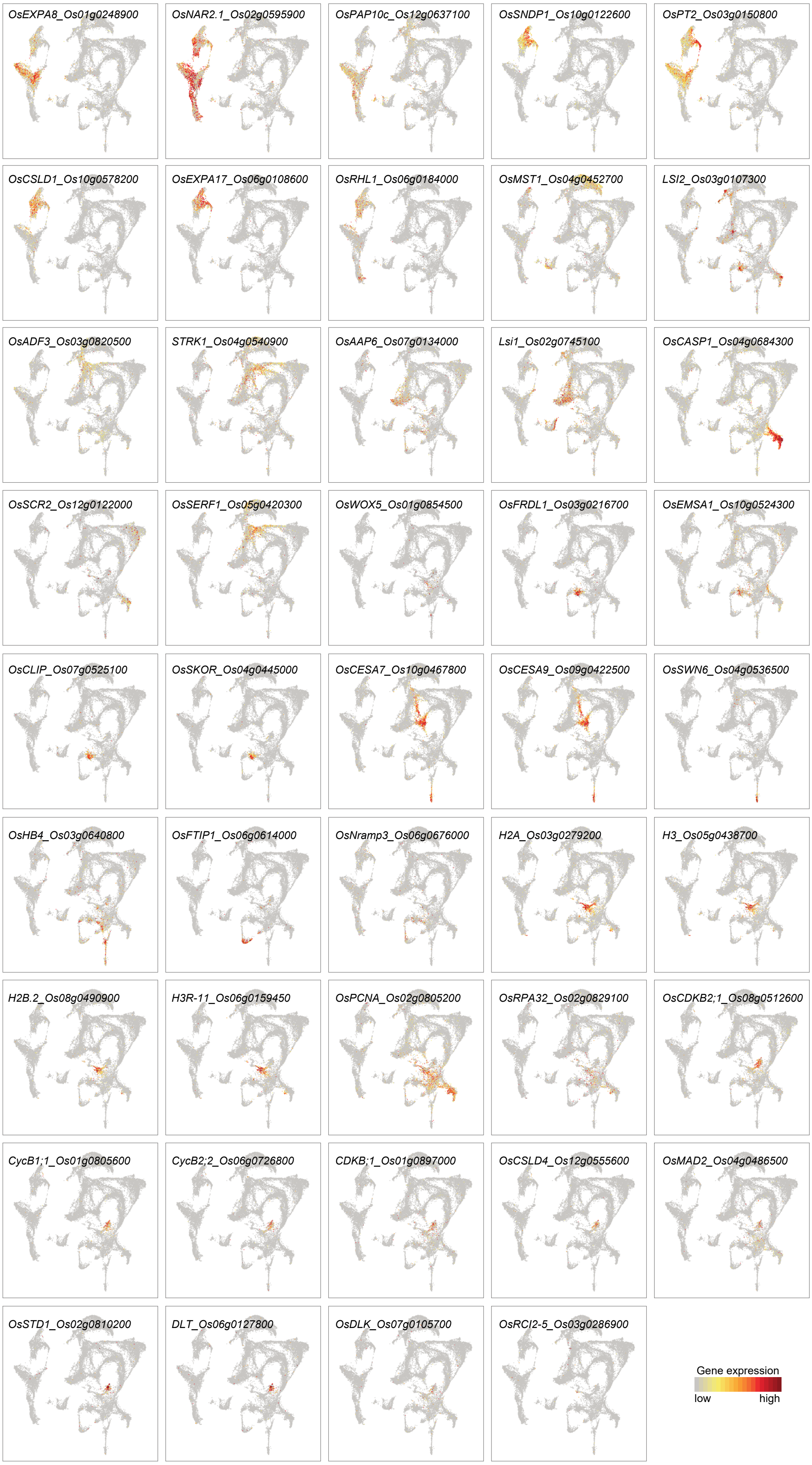
OsEXPA8_Os01g0248900
OsNAR2.1_Os02g0595900
OsPAP10c_Os12g0637100
OsSNDP1_Os10g0122600
OsPT2_Os03g0150800
OsCSLD1_Os10g0578200
OsEXPA17_Os06g0108600
OsRHL1_Os06g0184000
OsMST1_Os04g0452700
LSI2_Os03g0107300
OsADF3_Os03g0820500
STRK1_Os04g0540900
OsAAP6_Os07g0134000
Lsi1_Os02g0745100
OsCASP1_Os04g0684300
OsSCR2_Os12g0122000
OsSERF1_Os05g0420300
OsWOX5_Os01g0854500
OsFRDL1_Os03g0216700
OsEMSA1_Os10g0524300
OsCLIP_Os07g0525100
OsSKOR_Os04g0445000
OsCESA7_Os10g0467800
OsCESA9_Os09g0422500
OsSWN6_Os04g0536500
OsHB4_Os03g0640800
OsFTIP1_Os06g0614000
OsNramp3_Os06g0676000
H2A_Os03g0279200
H3_Os05g0438700
H2B.2_Os08g0490900
H3R-11_Os06g0159450
OsPCNA_Os02g0805200
OsRPA32_Os02g0829100
OsCDKB2;1_Os08g0512600
CycB1;1_Os01g0805600
CycB2;2_Os06g0726800
CDKB;1_Os01g0897000
OsCSLD4_Os12g0555600
OsMAD2_Os04g0486500
OsSTD1_Os02g0810200
DLT_Os06g0127800
OsDLK_Os07g0105700
OsRCI2-5_Os03g0286900
Gene expression
low
high

Supplementary Fig. 9 | Expression pattern of selected marker genes for cell cluster annotation. UMAP plot showing the expression levels of selected marker genes in each cell type. The marker genes for epidermal, exodermal, vascular cylinder, endodermal, pericycle, xylem, phloem and meristematic cells were shown, respectively. The full names and referenced of these selected genes are summarized in Supplementary Data 3.

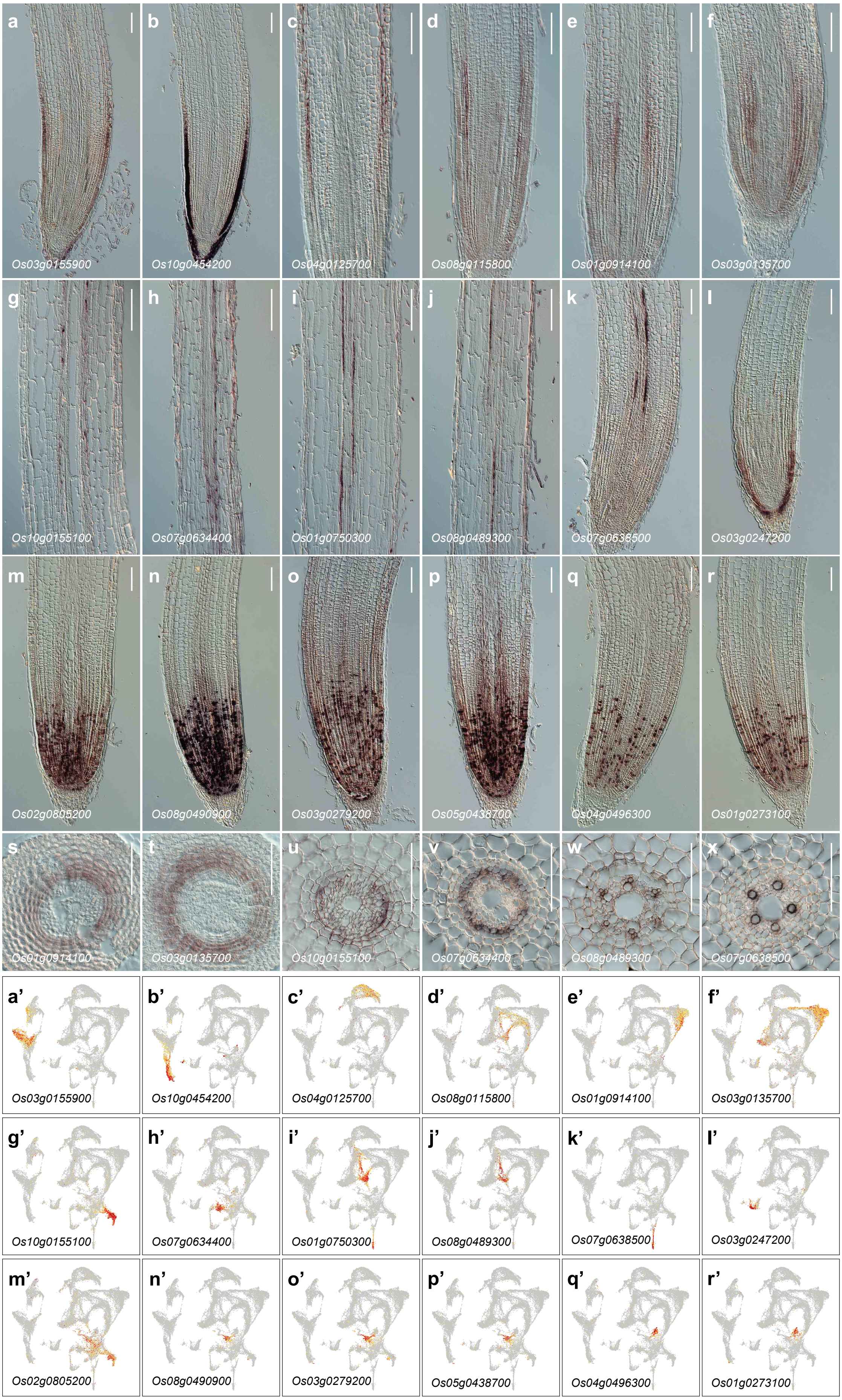
a
Os03g0155900
b
Os10g0454200
c
Os04g0125700
d
Os08g0115800
e
Os01g0914100
f
Os03g0135700
g
Os10g0155100
h
Os07g0634400
i
Os01g0750300
j
Os08g0489300
k
Os07g0638500
l
Os03g0247200
m
Os02g0805200
n
Os08g0490900
o
Os03g0279200
p
Os05g0438700
q
Os04g0496300
r
Os01g0273100
s
Os01g0914100
t
Os03g0135700
u
Os10g0155100
v
Os07g0634400
w
Os08g0489300
x
Os07g0638500
a’
Os03g0155900
b’
Os10g0454200
c’
Os04g0125700
d’
Os08g0115800
e’
Os01g0914100
f’
Os03g0135700
g’
Os10g0155100
h’
Os07g0634400
i’
Os01g0750300
j’
Os08g0489300
k’
Os07g0638500
l’
Os03g0247200
m’
Os02g0805200
n’
Os08g0490900
o’
Os03g0279200
p’
Os05g0438700
q’
Os04g0496300
r’
Os01g0273100

Supplementary Fig. 10 | Validation of cell types by *in situ* hybridization assays

The expression patterns of selected marker genes in root tissues using RNA *in situ* hybridization assays (a to x) and UMAP plots (a' to r'). We have examined over 30 cluster-specific genes. However, some of them did not give reliable signals (data not shown). Roots are longitudinally (a to r) or transversely (s to x) sectioned. Eight individual plants for each gene were observed. Scale bar, 100 µm.

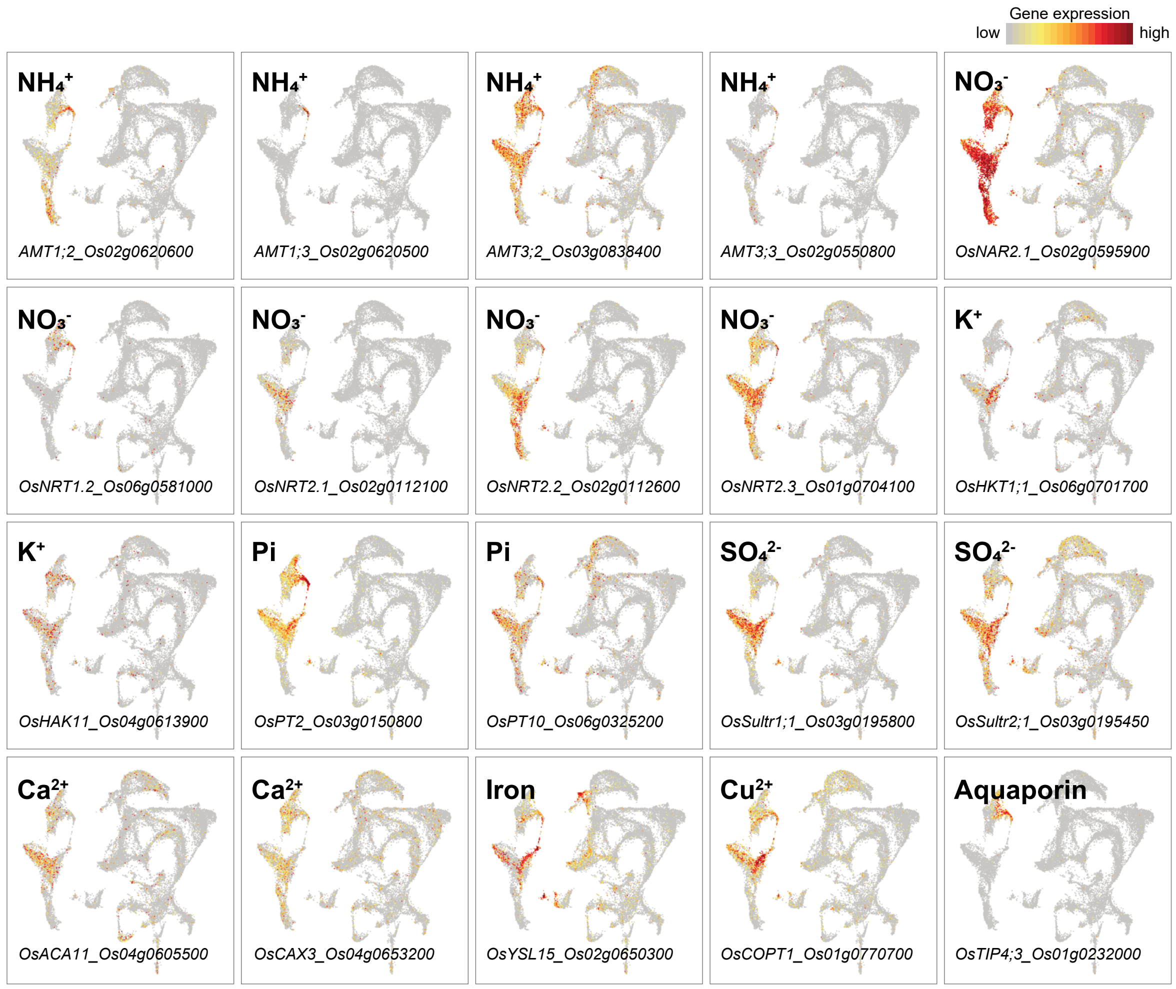

Supplementary Fig. 11 | Expression pattern of the genes involved in nutrition and ion assimilation. The expression levels of transporter genes were visualized on the UMAP plot. Please note that these transporter genes are highly enriched in the epidermal cell populations and exhibited cell-type specificity. For example, *OsTIP4;3* was predominately expressed in cluster 4. The full names and referenced expression pattern of selected genes are summarized in Supplementary Data 3.

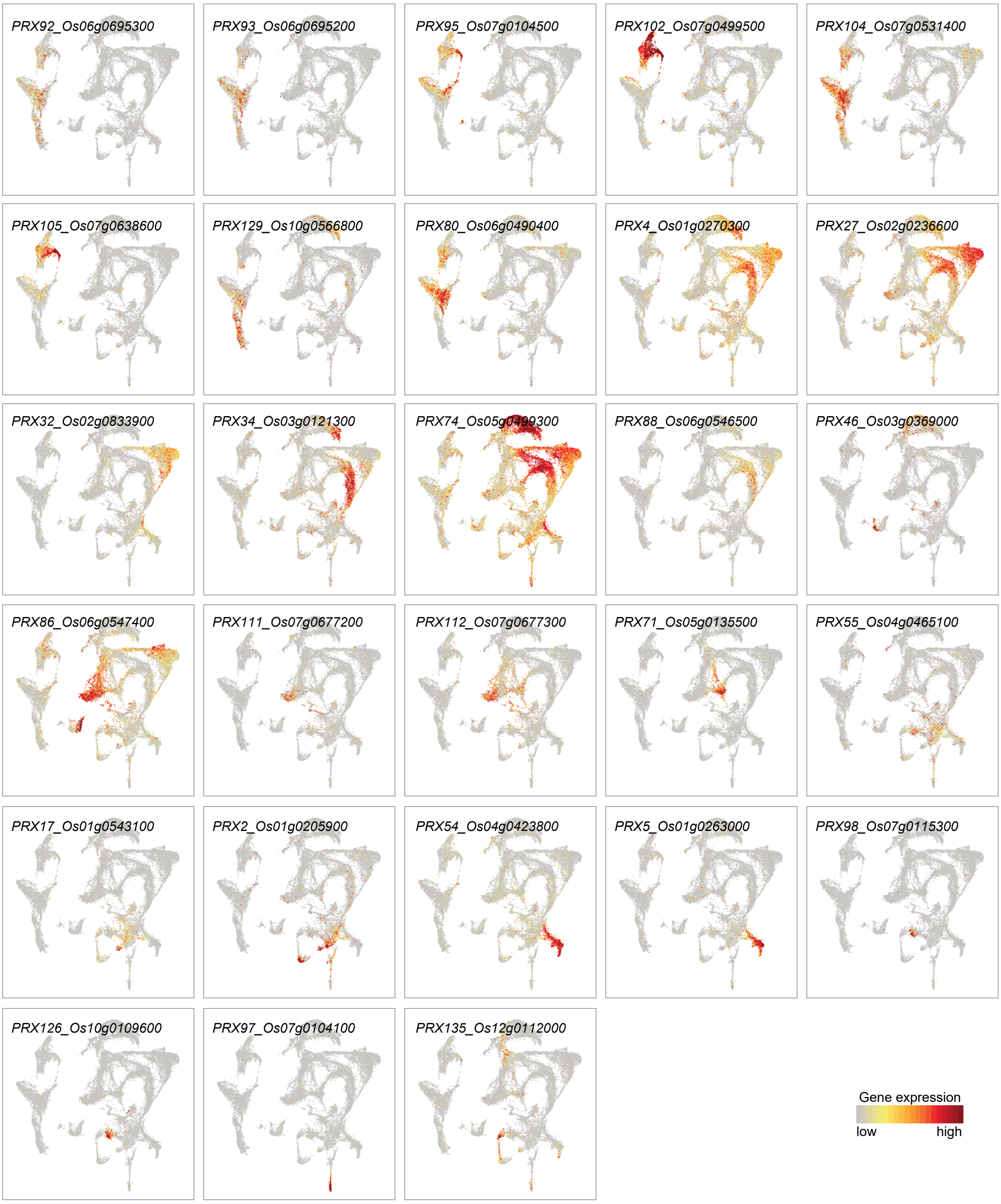

Supplementary Fig. 12 | Distinct expression pattern of peroxidases genes.

UMAP plot showing the expression pattern of *PRX* genes across rice root cell types. Please note that the expression of *PRX* genes are highly cell-type specific. For example, *PRX54* and *PRX5* were highly expressed in cluster 13 (endodermis), whereas *PRX86*, *PRX111* and *PRX112* were predominantly expressed in cluster 2 (endodermis). In contrast, *PRX27*, *PRX32* and *PRX74* and *PRX86* were detected

in cluster 0 (cortex). These expression patterns suggest that *PRXs* may exert different roles in distinct cell types.

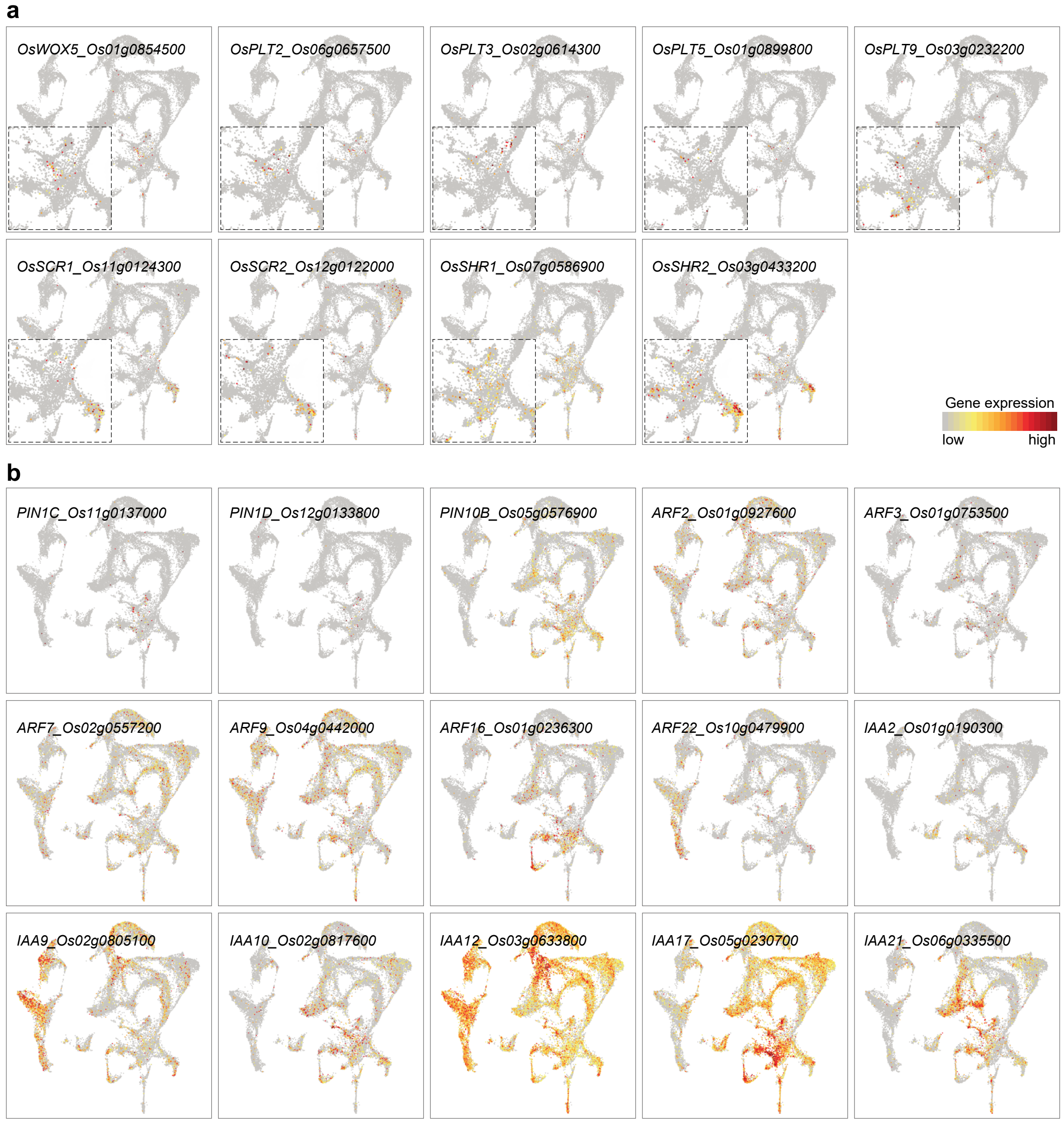

Supplementary Fig. 13 | Expression pattern of known genes involved in root development.

a, UMAP plot showing the expression pattern of *OsWOX5*, *OsPLTs*, *OsSCRs* and *OsSHRs* across rice root cell types. **b**, UMAP plot showing the expression pattern of auxin transport gene (*OsPINs*) and signaling factors (*ARFs* and *AUX/IAAs*) across rice root cell types.

Supplementary References

1 Hochholdinger, F. & Zimmermann, R. Conserved and diverse mechanisms in root development. *Curr Opin Plant Biol* **11**, 70-74, doi:10.1016/j.pbi.2007.10.002 (2008).

2 Mai, C. D. *et al.* Genes controlling root development in rice. *Rice* **7**, 30, doi:10.1186/s12284-014-0030-5 (2014).

3 Meng, F., Xiang, D., Zhu, J., Li, Y. & Mao, C. Molecular Mechanisms of Root Development in Rice. *Rice* **12**, 1, doi:10.1186/s12284-018-0262-x (2019).

4 Jun, N. *et al.* OsIAA23-mediated auxin signaling defines postembryonic maintenance of QC in rice. *Plant J* **68**, 433-442, doi:10.1111/j.1365-313X.2011.04698.x (2011).

5 Sarkar, A. K. *et al.* Conserved factors regulate signalling in Arabidopsis thaliana shoot and root stem cell organizers. *Nature* **446**, 811-814, doi:10.1038/nature05703 (2007).

6 Kamiya, N., Nagasaki, H., Morikami, A., Sato, Y. & Matsuoka, M. Isolation and characterization of a rice WUSCHEL-type homeobox gene that is specifically expressed in the central cells of a quiescent center in the root apical meristem. *Plant J* **35**, 429-441, doi:10.1046/j.1365-313x.2003.01816.x (2003).

7 Chu, H. *et al.* A CLE-WOX signalling module regulates root meristem maintenance and vascular tissue development in rice. *J Exp Bot* **64**, 5359-5369, doi:10.1093/jxb/ert301 (2013).

8 Suzaki, T., Yoshida, A. & Hirano, H. Y. Functional diversification of CLAVATA3-related CLE proteins in meristem maintenance in rice. *Plant Cell* **20**, 2049-2058, doi:10.1105/tpc.107.057257 (2008).

9 Fiers, M. *et al.* The 14-amino acid CLV3, CLE19, and CLE40 peptides trigger consumption of the root meristem in Arabidopsis through a CLAVATA2-dependent pathway. *Plant Cell* **17**, 2542-2553, doi:10.1105/tpc.105.034009 (2005).

10 Ito, Y. *et al.* Dodeca-CLE peptides as suppressors of plant stem cell differentiation. *Science* **313**, 842-845, doi:10.1126/science.1128436 (2006).

11 Colmer, T. D. Long-distance transport of gases in plants: A perspective on internal aeration and radial oxygen loss from roots. *Plant Cell Environ* **26**, 17-36 (2003).

12 Yamauchi, T. *et al.* Fine control of aerenchyma and lateral root development through

AUX/IAA- and ARF-dependent auxin signaling. *Proc Natl Acad Sci U S A* **116**, 20770-20775, doi:10.1073/pnas.1907181116 (2019).

13 Yamauchi, T. *et al.* Ethylene-dependent aerenchyma formation in adventitious roots is regulated differently in rice and maize. *Plant Cell Environ* **39**, 2145-2157, doi:10.1111/pce.12766 (2016).

14 Rebouillat, J. *et al.* Molecular Genetics of Rice Root Development. *Rice* **2**, 15-34 (2009).

15 Coudert, Y., Perin, C., Courtois, B., Khong, N. G. & Gantet, P. Genetic control of root development in rice, the model cereal. *Trends Plant Sci* **15**, 219-226, doi:10.1016/j.tplants.2010.01.008 (2010).

16 Johnson, A. A. *et al.* Spatial control of transgene expression in rice (Oryza sativa L.) using the GAL4 enhancer trapping system. *Plant J* **41**, 779-789, doi:10.1111/j.1365-313X.2005.02339.x (2005).

17 Jiao, Y. *et al.* A transcriptome atlas of rice cell types uncovers cellular, functional and developmental hierarchies. *Nat Genet* **41**, 258-263, doi:10.1038/ng.282 (2009).

18 Ding, W. *et al.* A transcription factor with a bHLH domain regulates root hair development in rice. *Cell Res* **19**, 1309-1311, doi:10.1038/cr.2009.109 (2009).

19 Kim, C. M., Han, C. D. & Dolan, L. RSL class I genes positively regulate root hair development in Oryza sativa. *New Phytol* **213**, 314-323, doi:10.1111/nph.14160 (2017).

20 Kim, C. M. *et al.* OsCSLD1, a cellulose synthase-like D1 gene, is required for root hair morphogenesis in rice. *Plant Physiol* **143**, 1220-1230, doi:10.1104/pp.106.091546 (2007).

21 Huang, J. *et al.* OsSNDP1, a Sec14-nodulin domain-containing protein, plays a critical role in root hair elongation in rice. *Plant Mol Biol* **82**, 39-50, doi:10.1007/s11103-013-0033-4 (2013).

22 Zhang, L. *et al.* OsPT2, a phosphate transporter, is involved in the active uptake of selenite in rice. *New Phytol* **201**, 1183-1191, doi:10.1111/nph.12596 (2014).

23 Lu, L. *et al.* OsPAP10c, a novel secreted acid phosphatase in rice, plays an important role in the utilization of external organic phosphorus. *Plant Cell Environ* **39**, 2247-2259, doi:10.1111/pce.12794 (2016).

24 Chen, G. *et al.* OsHAK1, a High-Affinity Potassium Transporter, Positively Regulates Responses to Drought Stress in Rice. *Front Plant Sci* **8**, 1885, doi:10.3389/fpls.2017.01885

(2017).

25 Chen, G. *et al.* Rice potassium transporter OsHAK1 is essential for maintaining potassium-mediated growth and functions in salt tolerance over low and high potassium concentration ranges. *Plant Cell Environ* **38**, 2747-2765, doi:10.1111/pce.12585 (2015).

26 Suenaga, A. *et al.* Constitutive expression of a novel-type ammonium transporter OsAMT2 in rice plants. *Plant Cell Physiol* **44**, 206-211, doi:10.1093/pcp/pcg017 (2003).

27 Liu, X. *et al.* Identification and functional assay of the interaction motifs in the partner protein OsNAR2.1 of the two-component system for high-affinity nitrate transport. *New Phytol* **204**, 74-80, doi:10.1111/nph.12986 (2014).

28 Lee, Y., Rubio, M. C., Alassimone, J. & Geldner, N. A mechanism for localized lignin deposition in the endodermis. *Cell* **153**, 402-412, doi:10.1016/j.cell.2013.02.045 (2013).

29 Wang, Z. *et al.* OsCASP1 Is Required for Casparian Strip Formation at Endodermal Cells of Rice Roots for Selective Uptake of Mineral Elements. *Plant Cell* **31**, 2636-2648, doi:10.1105/tpc.19.00296 (2019).

30 Yamaji, N. & Ma, J. F. Spatial distribution and temporal variation of the rice silicon transporter Lsi1. *Plant Physiol* **143**, 1306-1313, doi:10.1104/pp.106.093005 (2007).

31 Yokosho, K., Yamaji, N., Ueno, D., Mitani, N. & Ma, J. F. OsFRDL1 is a citrate transporter required for efficient translocation of iron in rice. *Plant Physiol* **149**, 297-305, doi:10.1104/pp.108.128132 (2009).

32 Liu, F. *et al.* Interactions of Oryza sativa OsCONTINUOUS VASCULAR RING-LIKE 1 (OsCOLE1) and OsCOLE1-INTERACTING PROTEIN reveal a novel intracellular auxin transport mechanism. *New Phytol* **212**, 96-107, doi:10.1111/nph.14021 (2016).

33 Kim, H. Y. *et al.* Differential gene expression of two outward-rectifying shaker-like potassium channels OsSKOR and OsGORK in rice. *Journal of Plant Biology* **58**, 230–235 (2015).

34 Song, S. *et al.* OsFTIP1-Mediated Regulation of Florigen Transport in Rice Is Negatively Regulated by the Ubiquitin-Like Domain Kinase OsUbDKgamma4. *Plant Cell* **29**, 491-507, doi:10.1105/tpc.16.00728 (2017).

35 Barros, J., Serk, H., Granlund, I. & Pesquet, E. The cell biology of lignification in higher plants.

Ann Bot **115**, 1053-1074, doi:10.1093/aob/mcv046 (2015).

36 Zhong, R. *et al.* Transcriptional activation of secondary wall biosynthesis by rice and maize NAC and MYB transcription factors. *Plant Cell Physiol* **52**, 1856-1871, doi:10.1093/pcp/pcr123 (2011).

37 Yoshida, K. *et al.* Engineering the *Oryza sativa* cell wall with rice NAC transcription factors regulating secondary wall formation. *Front Plant Sci* **4**, 383, doi:10.3389/fpls.2013.00383 (2013).

38 Itoh, J., Hibara, K., Sato, Y. & Nagato, Y. Developmental role and auxin responsiveness of Class III homeodomain leucine zipper gene family members in rice. *Plant Physiol* **147**, 1960-1975, doi:10.1104/pp.108.118679 (2008).

39 Schmidt, R. *et al.* Salt-responsive ERF1 regulates reactive oxygen species-dependent signaling during the initial response to salt stress in rice. *Plant Cell* **25**, 2115-2131, doi:10.1105/tpc.113.113068 (2013).

40 Zhou, Y. B. *et al.* The Receptor-Like Cytoplasmic Kinase STRK1 Phosphorylates and Activates CatC, Thereby Regulating H_2O_2 Homeostasis and Improving Salt Tolerance in Rice. *Plant Cell* **30**, 1100-1118, doi:10.1105/tpc.17.01000 (2018).

41 Huang, Y. C., Huang, W. L., Hong, C. Y., Lur, H. S. & Chang, M. C. Comprehensive analysis of differentially expressed rice actin depolymerizing factor gene family and heterologous overexpression of OsADF3 confers Arabidopsis Thaliana drought tolerance. *Rice* **5**, 33, doi:10.1186/1939-8433-5-33 (2012).

42 Pyronnet, S. & Sonenberg, N. Cell-cycle-dependent translational control. *Curr Opin Genet Dev* **11**, 13-18, doi:10.1016/s0959-437x(00)00150-7 (2001).

43 Hsu, Y. C., Li, L. & Fuchs, E. Transit-amplifying cells orchestrate stem cell activity and tissue regeneration. *Cell* **157**, 935-949, doi:10.1016/j.cell.2014.02.057 (2014).

44 Iijima, M., Morita, S. & Barlow, P. W. Structure and Function of the Root Cap. *Plant Production Science* **11**, 17-27 (2008).

45 Benfey, P. N. Defining the Path from Stem Cells to Differentiated Tissue. *Curr Top Dev Biol* **116**, 35-43, doi:10.1016/bs.ctdb.2015.12.002 (2016).

46 Santuari, L. *et al.* The PLETHORA Gene Regulatory Network Guides Growth and Cell Differentiation in Arabidopsis Roots. *Plant Cell* **28**, 2937-2951, doi:10.1105/tpc.16.00656 (2016).

47 Barbosa, I. C. R., Hammes, U. Z. & Schwechheimer, C. Activation and Polarity Control of PIN-FORMED Auxin Transporters by Phosphorylation. *Trends Plant Sci* **23**, 523-538, doi:10.1016/j.tplants.2018.03.009 (2018).

48 Israeli, A., Reed, J. W. & Ori, N. Genetic dissection of the auxin response network. *Nat Plants* **6**, 1082-1090, doi:10.1038/s41477-020-0739-7 (2020).

49 Roosjen, M., Paque, S. & Weijers, D. Auxin Response Factors: output control in auxin biology. *J Exp Bot* **69**, 179-188, doi:10.1093/jxb/erx237 (2018).

50 Strader, L. C. & Zhao, Y. Auxin perception and downstream events. *Curr Opin Plant Biol* **33**, 8-14, doi:10.1016/j.pbi.2016.04.004 (2016).

51 Korsunsky, I. *et al.* Fast, sensitive and accurate integration of single-cell data with Harmony. *Nat Methods* **16**, 1289-1296, doi:10.1038/s41592-019-0619-0 (2019).

52 Wang, Y., Huan, Q., Chu, X., Li, K. & Qian, W. Single-cell transcriptome analyses recapitulate the cellular and developmental responses to abiotic stresses in rice. *bioRxiv*, 2020.2001.2030.926329, doi:doi: https://doi.org/10.1101/2020.01.30.926329 (2020).
