# Supplementary material for: Single-cell transcriptome atlas and chromatin accessibility landscape reveal differentiation trajectories in the rice root
Source: Nat Commun. 2021 Apr 6;12:2053. doi: 10.1038/s41467-021-22352-4 (PMC8024345; doi:10.1038/s41467-021-22352-4)
Supplement: Supplementary file 3 — Descriptions of Additional Supplementary Files [file 41467_2021_22352_MOESM3_ESM.docx]

Descriptions of Additional Supplementary Files

**Supplementary Movie 1
Description:** A 360° view of the 3D UMAP plot.

**Supplementary Data 1
Description:** Cell Ranger reports.

**Supplementary Data 2
Description:** Cluster-enriched genes.

**Supplementary Data 3
Description:** The full names of the genes described in this study.

**Supplementary Data 4
Description:** GO analysis of 21 root cell clusters.

**Supplementary Data 5
Description:** Assignment of differentially accessible peaks.

**Supplementary Data 6
Description:** GO analysis of differentially accessible genes.

**Supplementary Data 7
Description:** One-to-one orthologs for cross-species comparison.

**Supplementary Data 8
Description:** Primers used in this study

**Supplementary Data 9
Description:** Conserved and divergent genes in rice and Arabidopsis root development.
