# Supplementary figures and images for: Single-cell transcriptome atlas and chromatin accessibility landscape reveal differentiation trajectories in the rice root
Source: Nat Commun. 2021 Apr 6;12:2053. doi: 10.1038/s41467-021-22352-4 (PMC8024345; doi:10.1038/s41467-021-22352-4)

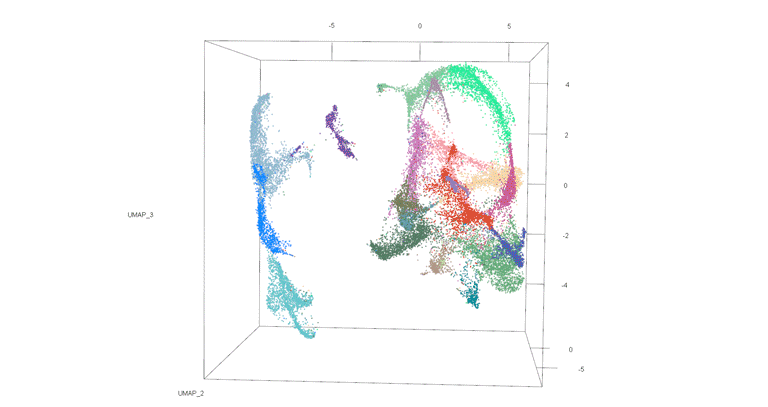

Supplement: Supplementary file 4 — Supplementary Movie 1 [file 41467_2021_22352_MOESM4_ESM.gif]
